# Cannabinoid receptor 1 knockout alleviates hepatic steatosis by downregulating perilipin 2

**DOI:** 10.1038/s41374-019-0327-5

**Published:** 2019-09-30

**Authors:** Karuna Irungbam, Yuri Churin, Tomomitsu Matono, Jakob Weglage, Matthias Ocker, Dieter Glebe, Martin Hardt, Alica Koeppel, Martin Roderfeld, Elke Roeb

**Affiliations:** 1 Department of Gastroenterology, Justus-Liebig-University, Giessen, Germany; 2 Institute for Surgical Research, Philipps University of Marburg, Marburg, Germany; 3 Institute of Medical Virology, National Reference Centre for Hepatitis B and D Viruses, Justus-Liebig-University, Giessen, Germany; 4 Central Biotechnical Facility, Justus-Liebig-University, Giessen, Germany; 5 Present Address: Department of Gastroenterology CBF, Translational Medicine Oncology, Charité University Medicine Berlin and Bayer AG, Experimental Medicine Oncology, Berlin, Germany

**Keywords:** Experimental models of disease, Proteolysis

## Abstract

The endocannabinoid (EC) system has been implicated in the pathogenesis of several metabolic diseases, including nonalcoholic fatty liver disease (NAFLD). With the current study we aimed to verify the modulatory effect of endocannabinoid receptor 1 (CB1)-signaling on perilipin 2 (PLIN2)-mediated lipophagy. Here, we demonstrate that a global knockout of the cannabinoid receptor 1 gene (CB1^−/−^) reduced the expression of the lipid droplet binding protein PLIN2 in the livers of CB1^−/−^ and hepatitis B surface protein (HBs)-transgenic mice, which spontaneously develop hepatic steatosis. In addition, the pharmacologic activation and antagonization of CB1 in cell culture also caused an induction or reduction of PLIN2, respectively. The decreased PLIN2 expression was associated with suppressed lipogenesis and triglyceride (TG) synthesis and enhanced autophagy as shown by increased colocalization of LC3B with lysosomal-associated membrane protein 1 (LAMP1) in HBs/CB1^−/−^ mice. The induction of autophagy was further supported by the increased expression of LAMP1 in CB1^−/−^ and HBs/CB1^−/−^ mice. LAMP1 and PLIN2 were co-localized in HBs/CB1^−/−^ indicating autophagy of cytoplasmic lipid droplets (LDs) i.e., lipophagy. Lipolysis of lipid droplets was additionally indicated by elevated expression of lysosomal acid lipase. In conclusion, these results suggest that loss of CB1 signaling leads to reduced PLIN2 abundance, which triggers lipophagy. Our new findings about the association between CB1 signaling and PLIN2 may stimulate translational studies analyzing new diagnostic and therapeutic options for NAFLD.

## Abstract (web summary)

The endocannabinoid (EC) system has been implicated in the pathogenesis nonalcoholic fatty liver diseases (NAFLD). Here the authors demonstrate that endocannabinoid receptor 1 (CB1) receptor knockout in vivo and pharmacologic antagonization of CB1 in cell culture decreases expression of lipid droplet binding protein perilipin 2, which might be an essential step in lipid breakdown. Thus, pharmacologic modulation of the CB1-perilipin 2 axis might represent a novel therapeutic approach for the treatment of steatosis.

## Introduction

Hepatic steatosis and especially its progressive form nonalcoholic steatohepatitis (NASH) are major health problems and increasing causes for liver cirrhosis and hepatocellular cancer [1]. Disturbances in lipid metabolism play a critical role in the progression of nonalcoholic fatty liver disease (NAFLD) [2]. Though the regulating mechanisms of lipogenesis and lipolysis in NAFLD are discussed to a considerable extent in previous studies, the etiology of the disease remains elusive [1]. So far, there is no approved drug for the treatment of NAFL and NASH in patients. The treatment consists of lifestyle modification (e.g., sustainable weight reduction plans and physical training), which seems to improve the metabolic conditions linked to NAFLD [1, 3–5].

It has been reported that endocannabinoids (ECs) widely participate in central and peripheral lipid metabolism by activating G protein-coupled cannabinoid receptors type 1 and type 2 (CB1 and CB2) [6]. ECs stimulate the appetite to increase energy intake, but also promote lipogenesis in peripheral tissues, such as the adipose tissue, liver, and skeletal muscles, thus leading to obesity and fatty liver disease [7]. The basal hepatic expression of CBs is faint, with low levels of CB2 receptors in Kupffer cells and of CB1 receptors in endothelial cells and hepatocytes [8]. ECs with diet-induced obesity have been associated with fatty liver, insulin resistance and other phenotypes [9]. CB1 receptors have been reported to play a role in the development of fatty liver in Zucker rats whereas the CB1 antagonist rimonabant reduced obesity-associated hepatic steatosis and inflammatory response [10]. CB1 knockout (CB1KO) mice are resistant to diet-induced obesity even though their total caloric intake is similar to that of wild type (WT) littermates, which became obese on the same diet [11]. Rimonabant was used for weight reduction in humans [12]. Moreover, the treatment of obese mice with rimonabant led to a transient reduction of food intake and a marked, but sustained reduction of body weight and adiposity of these animals [11]. Therefore, modulating the EC system in order to treat obesity and the associated metabolic disorders depicts a promising therapeutic concept [5]. However, the mechanism by which CB1 receptor deficiency or blockade increases energy expenditure has not yet been determined.

HBV associated steatosis has been reported from epidemiological and experimental studies [13]. Prevalence of histopathological steatosis in patients with chronic Hepatitis B is around 28% (12–76%) [14]. Transgenic mice overexpressing HBV surface proteins [15] developed hepatic steatosis [16]. Apart from steatosis, also other histopathological changes in the same transgenic model including inflammation, regenerative hyperplasia, ER stress, and associated unfolded protein response, which are based on hepatic accumulation of HBs, have been reported [17–19]. ER stress mediated response could be one of the reasons for hepatic steatosis [20].

Autophagy occurs as a response to starvation and energy depletion and is considered to be an alternative strategy for survival under shortage of energy supplies [21]. CB1 signaling influences autophagy, which might assist the cell in adjusting to different metabolic states. CB1 activity indeed alters the autophagic flux, and modulatory activity is exerted in a noncanonical mTOR and BECLIN1-independent manner [22]. Furthermore, ECs play a role in inducing autophagy in various cancer cell lines [23]. Recently, the contribution of autophagy to cytoplasmic lipid droplets (cLD) degradation has been identified [21]. The link between autophagy and cLDs arose from the observation that lysosomal acid lipase (LAL) deficiency leads to an accumulation of cLDs in various organs [24]. Further, in order to deliver cellular LDs to the lysosomes, autophagosomes sequestrate these LDs and fuse with lysosomes, which then leads to degradation of the LDs [25]. A connection between chaperone-mediated autophagy (CMA) and macroautophagy in the clearance of hepatic LDs has been reported recently [26]. Perilipin 2 (PLIN2) deficiency enhances autophagy and depletes hepatic TG [26]. In addition, various genes encoding autophagic proteins have been manipulated to confirm the importance of autophagy/lipophagy in the regulation of hepatic triacylglycerol levels [27]. With the current study we demonstrate that the cannabinoid receptor CB1 affected the development of hepatic steatosis by regulating PLIN2 expression in HBs transgenic mice.

## Material and methods

### Animals

Mice with a global CB1 receptor knockout deletion were purchased from the European Mouse Mutant Archive. Generation and characteristics of transgenic lineage C.B6J-Tg (Alb1HBV)44Bri (HBVTg/c) has been described previously [15, 18]. Hybrids of CB1^−/−^ crossbred with HBVTg/six mice strain having inbred C57BL/6 genetic background were generated. At age of 12, 26, and 52 weeks (*n* = 7–9) mice were weighed, anaesthetized by isofluran inhalation, and subsequently killed by cervical dislocation. The harvest was performed early in the morning in ad libitum fed state. Livers were weighed immediately after surgery and samples were preserved and stored at −80 °C until analysis.

Transgenic mice were maintained at the central animal laboratory of the Justus-Liebig-University Giessen under specified pathogen free conditions. This study was carried out in strict accordance as per the recommendations laid in the guide for the care and use of laboratory animals of the German law of animal welfare. The mice received humane care and all experiments were approved by the committee on the ethics of animal experiments of the Regierungspraesidium Giessen, Germany (approved no, GI 20/10 A5/2012 and 128/2014).

### Western blot

Liver lysates were prepared in 1× Laemeli buffer and boil at 95 °C for 10 min, and then briefly centrifuged for 5 min. After SDS-PAGE, the gel was transferred to nitrocellulose membrane and immunoblotting procedures were followed as per the standard protocol. Proteins recognition was done by using specific antibodies against AMPK (Genetex, GTX50863-100), Phospho-AMPK (Genetex, GTX130429-25), LC3B (Novus, NB100-2220), p62 (Proteintech, 18420-1-AP), PLIN2 (Proteintech, 15294-1-AP), PLIN4 (Merck, ABS526), PLIN3, PLIN5 (Progen: GP30S; GP31S), GAPDH (Proteintech,60004-1-Ig), and β actin (Santa Cruz, sc47778). The proteins were visualized using peroxidase-conjugated secondary antibodies and chemiluminescent reagent, developed on X-ray developer machine (AGFA, CP1000) using high sensitivity film (Hypersensitive film, Amersham). Alternatively, proteins were visualized using alkaline phosphatase conjugated secondary antibodies and developed with soluble 5-bromo-4-chloro-3-indolyl phosphate and nitro blue tetrazolium.

### Histochemical analyses of mouse liver

Immunohistochemistry (IHC) was performed using Impress Peroxidase/Alkaline Detection Reagents (Vector Laboratories) and antibodies specific for GLUT1 (Abcam, ab115730), LC3B (Proteintech, 18725-1-AP), /PLIN2 (Proteintech, 15294-1-AP), and LAL (Novus, NBPI-54155SS). Double immunostaining was performed as described previously [28]. Color reaction was developed with the VECTOR VIP Peroxidase Substrate Kit, (Vector Laboratories) or HighDef^®^ red IHC AP chromogen Enzo. Image quantification was performed using NIH ImageJ1.x software.

### Immunofluorescence microscopy

Paraffin-embedded liver sections were used for performing the immunofluorescence staining. The sections were deparaffinized as per the standard protocol. Unmasking was done by boiling the sample in 1× citrate buffer in microwave oven for 10 min. Blocking was done using 10% BSA, 5% goat serum, and 5% mouse IgG (MKB-2213, Vector Labs) in phosphate-buffered saline (137 mM sodium chloride, 3 mM potassium chloride, 7 mM disodium hydrogen phosphate, and 11 mM dipotassium hydrogen phosphate, pH 7.4). Primary antibodies used for double staining; anti-rabbit LC3B, 1:100 (Proteintech, 18725-1-AP), and anti-rabbit PLIN2, 1:200 (Proteintech, 15294-1-AP), anti-mouse LAMP1, 1:100 (Genetex, GT25212), p62, 1:100 (Proteintech, 18420-1-AP). Fluorochrome-conjugated secondary antibodies, Alexa-488 or 546 (Invitrogen,) were used. Nucleus was stained with DAPI. Cover slips were mounted on using Fluoromount^TM^ aqueous mounting medium (Sigma, F4680). All the images were acquired with Fluorescent microscope (Leica, Leitz, and DMR3) mounted with Nikon camera (Coolpix 5400) and prepared with Adobe Photoshop 7.0.

### Oil red O staining

Oil red O staining was performed as described previously [29]. Staining was assessed by bright-field microscopy.

### Thin layer chromatography

The lipid extraction from liver tissues was performed as described recently [30] with slight modifications. 20 mg of frozen liver tissue were weighed and then homogenized with of hexane/2-propanol 3:2(v/v) to a final volume 50 times the volume of the tissue sample (1 g in 50 ml of solvent mixture) for 1 h. After dispersion the whole mixture was centrifuged at 4 °C at 10,000 × *g* for 10 min. The supernatant was transferred to a new vial and dried under a nitrogen gas, resuspended in an appropriate volume of chloroform/methanol 2:1(v/v) and applied in equal amount onto pretreated, prewashed TLC plates. The standard (Nonpolar TLC standard, Bio trend Chemikalien, cat no.1130) and samples were applied onto the TLC plates and chromatographically separated with the solvent system containing hexane/diethylether/acetic acid (90:10:1(v/v/v) to the top of the plate. Detection and quantification was performed with charring method using N/10 sulfuric acids. Image was captured by gel doc analyzer (Biometra, Göttingen, Germany).

### Quantitative triglyceride estimation

Quantitative estimation of triglycerides from the liver samples was performed as per the protocol provided in Abcam Triglycerides estimation kit (ab65336).

### Real-time PCR

RNA isolation from the liver samples were performed using Direct-zol RNA extraction kit (Zymo research, Cat no. R2072). cDNA synthesis was done using iScript cDNA synthesis kit (BIO-RAD Cat no. 1708891) qRT-PCR was performed as described previously [31]. Primers were ordered from Microsynth (Switzerland). qPCR data were analyzed using ΔΔCt method [32]. Primers used: PLIN2 forward. 5-GACCTTGTGTCCTCCGCTTAT-3, reverse-5′-CAACCGCAATTTGTGGCTC-3′; β-actin forward: 5′-CAG CTT CTT TGC AGC TCC TT-3′, reverse: 5’-AGT CCT TCT GAC CCA TTC CC-3′.

### Cell culture and treatment with rimonabant

The AML12 liver cell line (kind gift from Prof. Ralf Weiskirchen, Aachen) was cultured up to 90% confluency for all the assay in a 1:1 mixture of Dulbecco’s modified Eagle’s medium and Ham’s F12 medium (Gibco, Grand Island, USA) with 0.005 mg/ml insulin, 0.005 mg/ml transferrin, 5 ng/ml selenium (ITS mixture, Gibco), 40 ng/ml dexamethasone (Sigma-Aldrich, St. Louis, MO), and supplemented with 10% fetal bovine serum (Gibco) at 37 °C in a humidified atmosphere with 5% CO_2_.

For in vitro assay, rimonabant and methanandamide were dissolved in DMSO and applicated in presence of 2% FBS for 48 h in presence and absence of oleic acid (OA) as per the indicated doses for each assay. For induction of steatosis in vitro, cells were treated with OA (Sigma-Aldrich, St. Louis, MO) at a final concentration of 200 µM.

### Statistics

All measurements were performed in technical triplicates. All results are expressed as means ± standard error of the mean. Significant differences between four groups were determined by one-way ANOVA with Tukey’s multiple comparisons test using to compare test groups. Differences between two groups were determined by Mann–Whitney’s *U* test. All statistical analyses were performed with GraphPad Prism software (v5.0; GraphPad Software Inc., San Diego, CA) and *p < 0.05, was considered statistically significant.

## Results

### CB1 knockout (CB1KO) reduced hepatic triglycerides and lipid droplet accumulation in HBs transgenic mice

**Fig. 1:**
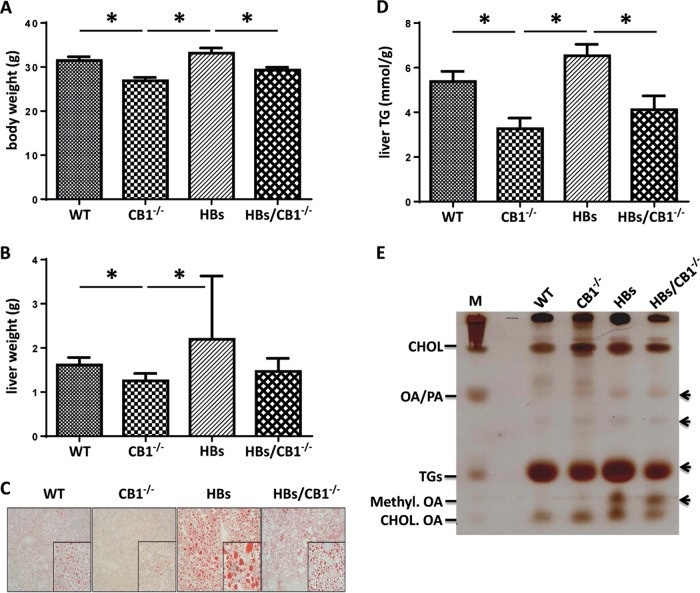
CB1 receptor knockout decreased body weight and hepatic lipid accumulation in mice. **a** Bar diagram showed significant reduction of body weight in 52-week-old CB1^−/−^ and HBs/ CB1^−/−^ mice in comparison with WT and HBs. Depicted are means ± SEM, *n* = 8–12. **b** Liver weight was reduced in CB1^−/−^-mice and enhanced in HBs transgenic mice. **c** Representative Oil red O staining of a 52-week-old mouse liver. **d** Liver triglyceride analysis demonstrated significant reduction of TGs level in the liver of 52-week-old CB1^−/−^ and HBS/CB1^−/−^ mice. *n* = 7–9. **e** Representative thin layer chromatography analysis demonstrated different total lipid profiles of 52-week-old mouse liver tissues. CHOL Cholesterol, OA/PA oleic acid/palmitic acid, TGs triglycerides

Maintenance of body weight and energy homeostasis depends on the coordinated regulation of appetitive behavior and peripheral energy metabolism [33]. CB1^−/−^ and HBs/CB1^−/−^ mice showed decreased body and liver weight compared with WT and HBs mice (Fig. 1a, b). As leanness has been associated with reduced steatosis, the effect of CB1KO on hepatic fat was analyzed. Oil red staining visualized increased hepatic steatosis in HBs transgenic mice (Fig. 1c). The CB1KO diminished the size of hepatic LDs in HBs/CB1^−/− ^mice. Quantification of liver TGs from liver samples of 52-week-old mice further demonstrated the reduction of TGs in CB1^−/−^ and HBs/CB1^−/−^ compared with WT and HBs mice (Fig. 1d).

Thin layer chromatography analysis of the total hepatic lipid extracts visualized the increased amounts of TGs in HBs and decreased TGs in CB1^−/−^ and HBs/CB1^−/−^ compared with WT (Fig. 1e). Interestingly, free fatty acids (OA/PA) were increased in CB1^−/−^, HBs, and HBs/CB1^−/−^ mice, which might be an effect of increased degradation of TGs. Due to the increase in free fatty acids in HBs transgenic mice, methyl ester derivatives of OA in liver samples of HBs and HBs/CB1^−/−^ mice were also increased (Fig. 1d). The cholesterol level remained unchanged. Taken together, these findings support the idea that the CB1KO led to the reduction of the size of hepatic LDs and TGs. In order to complement the features of NAFLD, we analyzed IL-1β and TNF-α as well as αSMA and desmin to assess CB1^−/−^-induced changes with regard to inflammation and fibrosis. Interestingly, inflammation and fibrosis were not significantly altered in HBs/CB1^−/−^ in comparison with HBs mice (Supplementary Figs. 1 and 2). Nevertheless, αSMA expression was increased in HBs transgenic mice in comparison with WT and CB1^−/−^mice and at least normalized by trend in HBs/CB1^−/−^ (Supplementary Fig. 2A). Serum aminotransferases ALT and AST were not regulated between HBs and HBs/CB1^−/− ^mice (not shown).

### CB1 knockout reduced PLIN2 expression

**Fig. 2:**
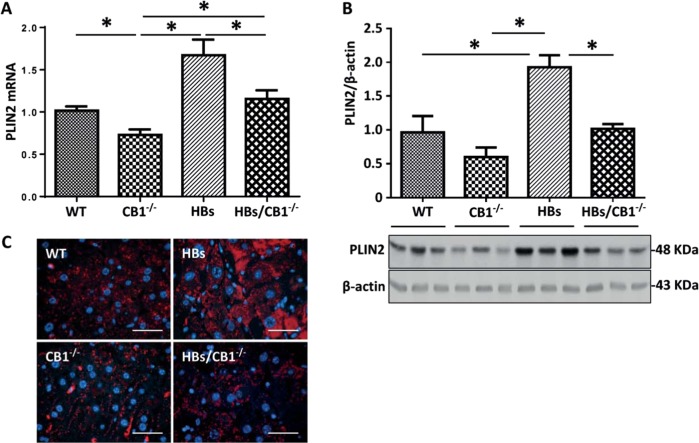
CB1 knockout reduced hepatic PLIN2 in HBs transgenic mice. **a** Quantitative real-time PCR analysis of PLIN2 revealed reduced PLIN2 mRNA in CB1^−/−^ and HBS/CB1^−/−^-mice. Data were normalized to β-actin as reference gene and shown as relative expression (ΔΔCt). **b** Western blot analysis of total protein lysates from the liver of 52-week-old mice demonstrates reduced PLIN2 protein levels in HBs/CB1^−/− ^mice. **c** Immunofluorescence analysis of paraffin-embedded liver sections from 52-week-old mice was performed using specific anti-PLIN2 antibody (red). Nuclei were stained with DAPI (blue). Original magnification ×1000, bar 40 µm

**Fig. 3:**
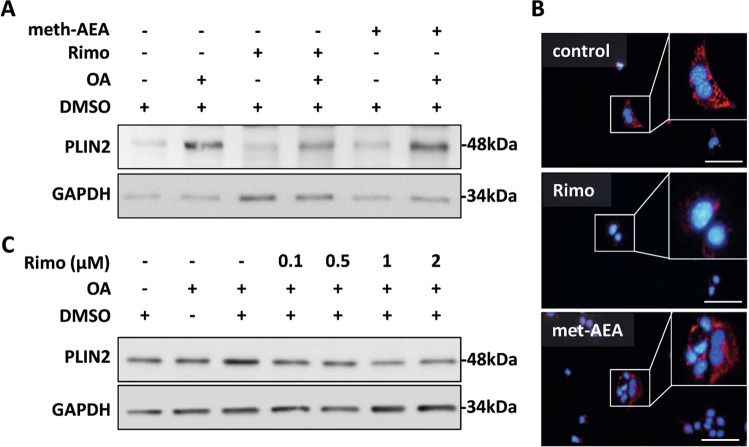
Pharmacologic antagonization of CB1 reduced PLIN2 expression in cell culture **a** AML12 cells were treated with rimonabant (rimo) or methanadamide (meth-AEA) at 1 µM final concentration for 48 h in the absence or presence of oleic acid (200 µM). Western blot analysis demonstrated enhanced PLIN2 expression with meth-AEA and reduced PLIN2 expression with rimo-treatment. Equal protein loading was confirmed using anti-GAPDH antibody. A representative Western Blot is shown. **b** Immunofluorescence analysis of PLIN2 (red) in AML12 cells treated with rimonabant or meth-AEA. Nuclei were stained with DAPI (blue). Representative stainings are shown. Original magnification ×400, bar 100 µm. **c** Dose dependent decrease in PLIN2 expression in AML12 cells treated with rimonabant at different doses (0.1, 0.5, 1, and 2 µM), respectively. in the presence of oleic acid (200 µM). Cell lysates were analyzed by western blot using specific anti-PLIN2 antibodies. Equal protein loading was confirmed using an anti-GAPDH antibody

The interesting changes in the shape and size of hepatic LDs in HBs/CB1^−/−^ mice raised the question, whether the mechanism of LD formation might be affected by the CB1KO. Perilipins are the major cLD-associated proteins which are involved in intracellular LD formation. The protein family consists of five members [34]. Among them, PLIN2 represents a constitutively and ubiquitously expressed protein that is used as a marker of LDs [26]. We observed lower expression levels of PLIN2 in the livers of CB1^−/−^ and HBs/CB1^−/−^ in comparison with WT and HBs mice (Fig. 2a–c). PLIN2 mRNA level was decreased in CB1^−/−^ and HBs/CB1^−/−^ compared with WT and HBs mice, respectively (Fig. 2a). This reduction of PLIN2 correlated with the reduction of TGs (Fig. 1). Western blot analysis and densitometric analysis demonstrated similar results on protein level. Immunostaining visualized the decreased PLIN2 expression in hepatocytes of HBs/CB1^−/−^ compared with HBs mice (Fig. 3c). The hepatic expression of other PLIN family members like PLIN3 and PLIN5 had been described before in the context of lipid metabolism [35] and was therefore also analyzed by western blot (Supplementary Fig. 2). PLIN3 protein expression was upregulated in HBs/CB1^−/−^ while PLIN4 was downregulated in HBs and HBs/CB1^−/−^ similarly. PLIN5 protein expression was decreased in the liver of HBs mice in comparison with HBs/CB1^−/−^ (Supplementary Fig. 3).

Analyses of PLIN2 expression in AML12 cells treated with the specific CB1 inhibitor rimonabant (Rimo) and agonist methanadamide (meth-AEA) were performed to prove the regulation of PLIN2 by CB1 mechanistically. Immunoblotting and immunofluorescence analysis were performed showing that inhibiting CB1 caused a decrease in PLIN2 expression in comparison with both vehicle control and meth-AEA (Fig. 3a, b). PLIN2 expression was also dose-dependently downregulated by Rimo in AML12 cells after OA treatment when compared with vehicle control (DMSO with OA) (Fig. 3c). Taken together, in vivo and in vitro experiments demonstrated hepatocellular reduction of PLIN2 expression by both CB1 receptor knockout and antagonization, respectively.

### CB1 knockout elevated autophagy in the liver of HBs transgenic mice

**Fig. 4:**
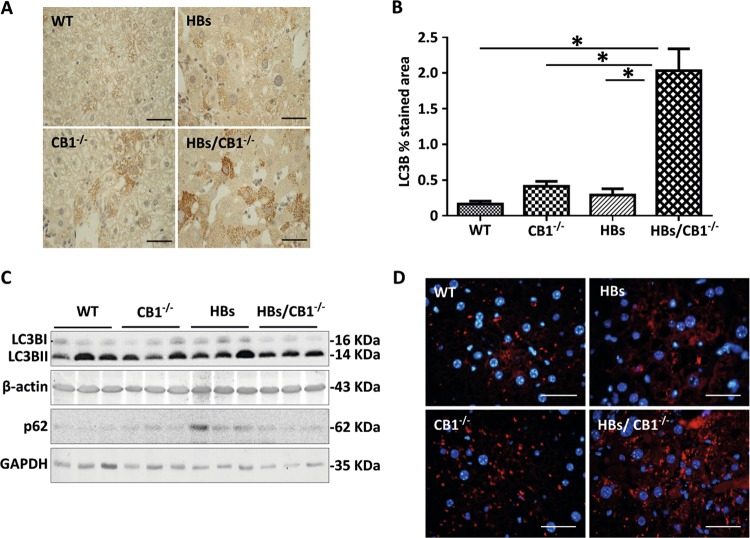
CB1 knockout elevated autophagy in the liver of HBs transgenic mice. **a** Representative immunohistochemical analysis of paraffin-embedded liver sections from 52-week-old mice was performed using anti-LC3B antibody. Original image magnification ×1000, bar 40 µm. **b** Quantification of LC3B puncta assessed with ImageJ software and expressed as % of LC3B staining/field. **c** Western blot analysis of lysates from 52-week-old mice was performed using specific anti-LC3B and anti-p62 antibodies. **d** Representative immunofluorescence analysis of paraffin-embedded liver sections of 52-week-old mice was performed using specific anti-LAMP1 antibodies (red). Nuclei were stained with DAPI (blue). Magnification ×1000, bar 40 µm

Autophagy is a response to starvation or energy depletion, and it is considered to be an alternative strategy for survival under such circumstances [21]. Therefore, we investigated whether there might be an enhancement of autophagy due to CB1KO. The expression levels of the proteins LC3B and p62 which are classical markers for autophagy were analyzed by western blotting [36]. LC3B immunostaining was significantly increased in HBs/CB1^−/−^ indicating enhanced autophagosome-formation (Fig. 4a, b). Western blotting demonstrated that LC3B protein expression was reduced by trend in HBs/CB1^−/−^ as compared with HBs transgenic mice (Fig. 4c and Supplementary Fig. 4A) possibly suggesting an autophagic flux. A temporal western blot analysis of total liver protein from HBs and HBs/CB1^−/−^ mice showed that LC3B II levels were increased in HBs/CB1^−/−^ compared with HBs at 12 and 26 weeks (Supplementary Fig. 4B). Interestingly, LC3B II levels were reduced after 52 weeks (Supplementary Fig. 4B and Fig. 4c). This decrease in elder animals might be due to temporally manifested stronger nutrient stress mediated by combined effects of CB1KO and HBs expression in hepatocytes [22]. Furthermore, LC3B II protein expression was also induced by rimonabant treatment in cell culture (data not shown). Hepatic p62 protein expression was decreased in HBs/CB1^−/−^ mice as compared with HBs mice (Fig. 4c). The staining of lysosomal-associated membrane protein 1 (LAMP1) revealed an enhanced expression of LAMP1 in CB1^−/−^ and HBs/CB1^−/−^ in comparison with WT and HBs mice (Fig. 4d).

**Fig. 5:**
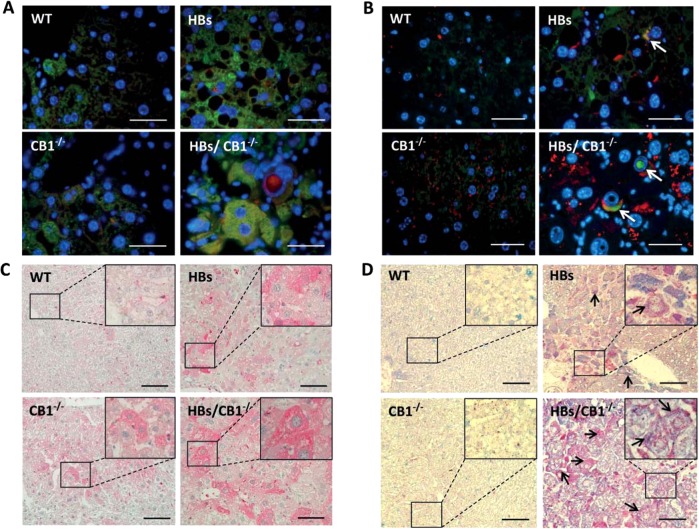
**a** Double immunofluorescence analysis of paraffin-embedded liver sections of 52-week-old mice was performed using specific anti-LC3B (green) and LAMP1 (red) antibodies. Nuclei were stained with DAPI (blue). Colocalization of these two proteins appears in yellow. Magnification ×1000 Scale bar 40 µm. **b** Representative double immunofluorescence analysis of paraffin-embedded liver sections from 52-week-old mice was performed using anti-p62 (green) and anti-LAMP1 (red). Arrowheads indicate colocalization of these proteins shown in yellow color and p62 accumulation in nuclear inclusion. Original image magnification ×1000, bars 40 µm. **c** Representative immunohistochemical analysis of paraffin-embedded liver sections from 52-week-old mice was performed using specific anti-LAL (lysosomal acid lipase) antibody. Original magnification ×200 and ×1000, bar 100 µm. **d** Representative immunohistochemical analysis of PLIN2 (blue) and LAMP1 (red). Arrows show costained areas (purple color). Original magnification ×200 and ×1000, bar 100 µm

Double immunofluorescence staining using anti-LC3B and anti-LAMP1 antibodies showed enhanced colocalization in HBs/CB1^−/−^ mice as compared with HBs mice (Fig. 5a). Moreover, double immunofluorescent staining using anti-p62 and anti-LAMP1 antibodies showed a partial colocalization of p62 with LAMP1 in HBs/CB1^−/−^ (Fig. 5b). Interestingly, p62 was also localized within the nuclear inclusions (Fig. 5b) and therefore protected from degradation by autophagy.

LAL is an acidic hydrolytic enzyme, which is responsible for lipid digestion. Its expression was assessed as an indicator of autophagy-mediated digestion of LDs [25]. Immunostaining of LAL revealed an increased accumulation of LAL protein in hepatocytes of HBs and CB1^−/−^ mice compared with WT mice (Fig. 5c). The strongest accumulation of LAL protein was observed in hepatocytes of HBs/CB1^−/−^ mice. To further confirm specific autophagy of lipids, immunohistochemical analyses of LAMP1 with PLIN2 were performed. We observed a stronger colocalization of LAMP1 and PLIN2 in HBs/CB1^−/−^ than in WT or HBs (Fig. 5d). Taken together, the lack of CB1 signaling enhanced the autophagic flux leading to an autophagy-mediated lipolysis of TGs in hepatocytes of HBs transgenic mice.

### CB1KO suppressed hepatic lipogenesis

**Fig. 6:**
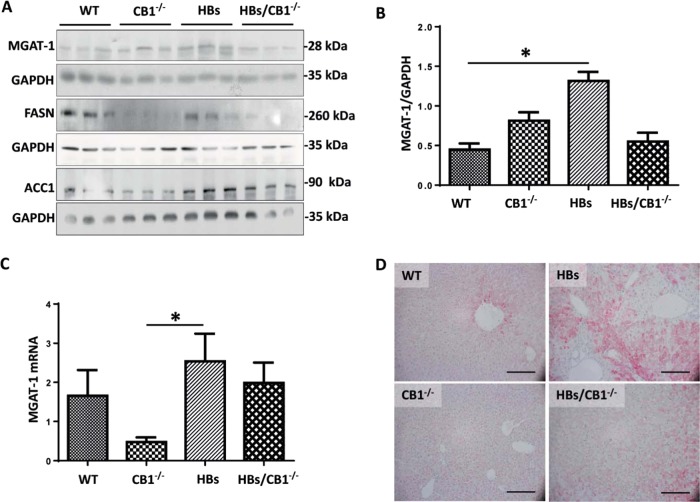
CB1 knockout induced suppression of lipogenesis in HBs transgenic mice. **a** Western blot analysis of liver lysates of 52-week-old mice was performed using specific anti-MGAT1, anti-FASN, and anti-ACC1 antibodies. Equal protein loading was controlled by anti-GAPDH and anti-β-actin antibodies, respectively. **b** Bar graph representing normalized MGAT1 protein expression to GAPDH. Statistic, one-way ANOVA, Kruskal–Wallis test (*P* = 0.004), Dunnet’s multiple comparison test were performed. **c** Quantitative real-time PCR (qRT-PCR) analysis of MGAT1 transcript was performed. The amount of RNA in the different samples is normalized to β-actin as reference gene and shown as relative expression (ΔΔCt). Statistical analysis was performed in GraphPad, One-way ANOVA (Kruskal–Wallis test, *P* = 0.0053). Dunn’s multiple comparison test was used to compare among different group. **d** Representative immunohistochemistry of paraffin section using specific DGAT1 antibody. Magnification, ×200 bar 200 µm

Disturbance of hepatic glucose uptake has been described as an indicator of metabolic stress [37] and lipogenesis. Immunohistochemical analysis revealed an increased localization of glucose transporter GLUT1 and GLUT2 in plasma membranes of hepatocytes in HBs/CB1^−/−^ compared with HBs (Supplementary Fig. 4A, B), suggesting a change in hepatic energy metabolism. Overall GLUT1 protein expression and mRNA level remained unchanged (data not shown). It has been reported that CB1KO leads to increased hepatic AMPK activation [9]. Western blot analysis of phospho-AMPK just demonstrated a tendential activation of AMPK in CB1KO in comparison with WT while this effect was abolished in HBs/CB1^−/−^ (Supplementary Fig. 5B). As the activation of AMPK may inhibit various anabolic pathways and stimulates catabolic pathways to restore the energy homoestasis [38], we explored the effect of CB1KO on lipogenesis. Fatty acid synthase and Acetyl-CoA-Carboxylase were decreased in CB1^−/−^ in comparison with HBs and WT (Fig. 6a). The transcriptional expression level of peroxisome proliferator-activated receptor gamma (PPARγ) was reduced in CB1KO and HBs/CB1^−/−^ in comparison with WT and HBs as shown in Supplementary Fig. 5D. Monoacylglycerol O-acyltransferase 1 (MGAT1) was significantly increased in HBs in comparison with WT whilst it was downregulated in HBs/CB1^−/−^ in comparison with HBs (Fig. 6a, b). Similarly, the transcriptional level of MGAT1 was also upregulated in HBs in comparison with CB1^−/−^ (Fig. 6c). IHC visualized the decrease in DGAT1 protein expression in HBs/CB1^−/−^ in comparison with HBs (Fig. 6d). In summary, CB1KO suppressed hepatic lipogenesis and TG synthesis in HBs/CB1^−/−^ mice.

## Discussion

In the current study, we investigated the impact of CB1 receptor knockout on fat accumulation in the livers of HBs transgenic mice which spontaneously develop hepatic steatosis [16]. EC signaling is known to regulate appetite, energy balance, and metabolic processes through both central and peripheral pathways [33]. ECs mediate their effects on energy and lipid metabolism by activating CB1 or CB2 receptors. Several studies using genetic deletion mutants or pharmacological antagonization of CB1 receptor have demonstrated anorectic effects that improved obesity as well as hepatic steatosis and metabolic disturbances [39]. It has been shown that CB1^−/−^ protects from steatosis in alcoholic liver injury, which provoked the suggestion that CB1 receptor activation aggravates SREBP-1c-mediated steatosis after ethanol intake [40]. Those data confirm the current findings. Our data also demonstrate the reduction of body weight, liver weight, and liver TGs in murine steatosis (Fig. 1a–e), thus confirming the previous findings.

The effect of CB1KO on the reduction of LD-associated PLIN2 expression has not been described previously. Whole body knockout of PLIN2 decreased the hepatic triglyceride level and protected against diet-induced obesity, and liver steatosis [41]. Protection against fatty liver in normal adipogenesis was documented in mice lacking adipose differentiation-related protein, another name of PLIN2 [42]. The reduction of PLIN2 expression by antisense oligonucleotide treatment led to decreased hepatic lipid accumulation [43]. Furthermore, a PLIN2 liver-specific ablation alleviates diet-induced hepatic steatosis and inflammation [44]. Herein, a phosphatidylethanolamine N-methyltransferase-mediated mechanism that involves compensatory changes in proteins involved in phospholipid remodeling, inflammation, and ER stress has been suggested to alleviate diet-induced NASH. All these studies support an important role for PLIN2 as a target for NASH therapy. Here we have demonstrated that the hepatic PLIN2 expression was reduced in the livers of CB1^−/−^ and HBs/CB1^−/−^ mice (Fig. 2a–c). In order to show that the hepatic changes of Perilipin 2 expression were at least in part a direct result of abolishing CB1 in liver, and not secondary effects of the reduced food intake and body weight phenotype associated with the loss of CB1 in CNS, pharmacologic CB1 antagonization was performed to demonstrate the effect in cell culture. Very interestingly, loss of hepatic PLIN2 was further shown to be associated with minor increases in PLIN3 and PLIN5 levels in CB1^−/−^ and HBs/CB1^−/−^ mice (Supplementary Fig. 3 A, B). The increase in PLIN3 might possibly be a compensatory mechanism regulating LD accumulation in the absence of PLIN2 [45]. PLIN5 is most highly expressed in oxidative tissues such as skeletal muscle, and considered to promote fat oxidation [46]. Therefore, the minor changes in PLIN5 might suggest an increased oxidative degradation of TGs in CB1KO mice.

Previous studies reported that cannabinoids and activation of cell surface cannabinoid receptors could bind directly and indirectly to PPARs [47]. PLIN2 can be regulated through PPARα and PPARγ [46]. In the current study PPARγ was downregulated in CB1KO and HBs/CB1 mice suggesting to be one of the mechanisms for CB1KO mediated regulation of PLIN2 expression (Supplementary Fig. 5D). Moreover, the downregulation of PLIN2 by CB1KO further leads to suppression of lipogenesis and TGs synthesis (Fig. 6) as described previously [48]. Similarly, the reduced expression of PLIN2 in AML12 following rimonabant treatment strengthens the hypothesis of the direct association between CB1 signaling and PLIN2 in hepatocytes (Fig. 3a–c). Hence, altering the expression level of PLIN2 could be a mechanism of CB1-mediated regulation of hepatic lipid metabolism. Furthermore, the depletion of PLIN2 resulted in reduced food intake in response to HFD feeding [42]. Food intake was also lower in CB1^−/−^ [49]. Since the global CB1KO was used in our study, we could not rule out the possibility that the cannabinoid receptor regulated food intake also affected PLIN2 expression. However, the cell culture experiments clearly demonstrated a reduction of PLIN2 expression by CB1 antagonization and an induction of PLIN2 expression by the CB1 activator meth-AEA. Therefore, it is likely that CB1KO regulates PLIN2 expression which further supports our hypothesis that PLIN2 was regulated by the CB1KO in murine liver. It was previously shown that LDs can be selectively sequestered in autophagosomes and delivered to lysosomes for degradation by LALs—a process known as “lipophagy” [21]. PLIN2, being the major LDs associated protein, protects and stabilizes LDs, provides a “shielding effect” and modulates lipase’s accessibility to TG [50]. Removal of the LD surface proteins, PLIN2 by CMA (chaperon mediated autophagy) represents the preliminary step that helps to open up the lipid components of LDs core for degradation by lipophagy or cytosolic lipase. Moreover, it has been previously reported that downregulation of PLIN2 stimulates the breakdown of TGs via autophagy [26]. Therefore, downregulation of PLIN2 could be considered as a pivotal step promoting autophagic lipolysis of LDs.

AMPK (AMP-activated protein kinase) is a sensor of energy status that responds to the increase of AMP or ADP cellular concentrations to maintain cellular energy homeostasis [51]. Although we could demonstrate a tendential increase in phosphor-AMPK in the livers of CB1^−/−^, AMPK was not activated in HBs/CB1^−/−^ mice (Supplementary Fig. 5C). Further, it has been reported that stimulation of AMPK is associated with enhancement of GLUT1-mediated glucose transport [52]. Our study demonstrated increased hepatic membrane localization of GLUT1 (supplementary Fig. 5A). Changes in GLUT1 conformation and cellular localization seem to be part of an adaptive host response to maintain adequate cellular nutrition and energy levels [53]. The production of endogenous HBV surface proteins in the hepatocytes of HBs mice might induce additional cellular stress, which could explain the higher surge of energy as shown by increased GLUT1 localization on plasma membrane. Interestingly, GLUT1 expression could be regulated by hypoxia [54]. In the livers of HBs/CB1^−/−^ mice GLUT1 was not regulated on mRNA and protein levels (data not shown) that excluded any effect of hypoxia on GLUT1 in our model. Recent studies demonstrated that CB1KO can modulate the autophagic flux in a noncanonical mTOR and BECLIN1-independent manner [22]. Autophagic flux implies autophagic degradation activity [36]. So far, a reliable biological markers used for identifying autophagic flux are the LC3B II turnover or SQSTM1/p62 degradation. Proofing this, however, imposes a challenge in an experimental set up to access the magnitude of autophagic flux especially in a steady state condition [55]. Therefore, we demonstrated increased autophagosomes formation as depicted by increased LC3B puncta and increased colocalization of LC3B and LAMP1. Moreover, immunoblotting showed decreased LC3B II protein levels in HBs/CB1^−/−^ which could be an effect of enhanced autophagic flux progression (Fig. 4a–d). The p62 protein expression level decreased in the liver of HBs/CB1^−/−^ compared with HBs mice (Fig. 4c). However, we demonstrated partial colocalization of p62 with LAMP1 that indicated autophagic degradation of p62 marked proteins and also p62 accumulation in nuclear inclusions (Fig. 5b). The transgenic model of HBV surface antigens exhibited nuclear inclusions in hepatocytes [56]. The fraction of nuclear inclusion localized p62 might be protected from degradation thus providing a possible explanation for similar expression levels of p62 in the livers of WT and HBs/CB1^−/−^ mice (Fig. 5b). In addition, the expression of LAMP1 has been reported as a marker to assess autophagic flux [57]. Hepatic LAMP1 protein expression was increased in our model (Fig. 5a) further indicating autophagic flux mediated by CB1KO. Furthermore, expression of acidic lipases, LAL (lysosomal-associated lipase), is assessed as an indicator of autophagy-mediated digestion of LDs [21]. LAL was upregulated in the livers of HBs/CB1^−/−^ mice that indicated enhanced lipolysis (Fig. 5c). In addition, the partial localization of LAMP1 and PLIN2 suggests the induction of lipophagy (Fig. 5d). Taken together, we demonstrated several markers that suggest enhanced autophagic flux and autophagic mediated lipolysis in HBs/CB1^−/−^. Finally, LD-associated lipases (ATGL) were not regulated by CB1^−/−^ (not shown) but we cannot exclude the possibility that any other lipolytic mechanisms could play a role in the effects observed.

In conclusion, we demonstrated that the CB1 receptor knockout in vivo and pharmacologic antagonization of CB1 in cell culture decreased PLIN2 expression, which might be an essential step in lipid breakdown (schematically summarized in Supplementary Fig. 6). Thus, pharmacologic modulation of the CB1-PLIN2 axis might represent a novel therapeutic approach for the treatment of steatosis.

## Supplementary information

Cannabinoid receptor 1 knockout alleviates hepatic steatosis by down regulating Perilipin 2-supplemental material
