# Supplementary material for: Cannabinoid receptor 1 knockout alleviates hepatic steatosis by downregulating perilipin 2
Source: Lab Invest. 2019 Sep 30;100(3):454–65. doi: 10.1038/s41374-019-0327-5 (PMC7044114; doi:10.1038/s41374-019-0327-5)
Supplement: Supplementary file 1 — Cannabinoid receptor 1 knockout alleviates hepatic steatosis by down regulating Perilipin 2-supplemental material [file 41374_2019_327_MOESM1_ESM.pdf]

Supplemental figure S1: Inflammatory cytokines

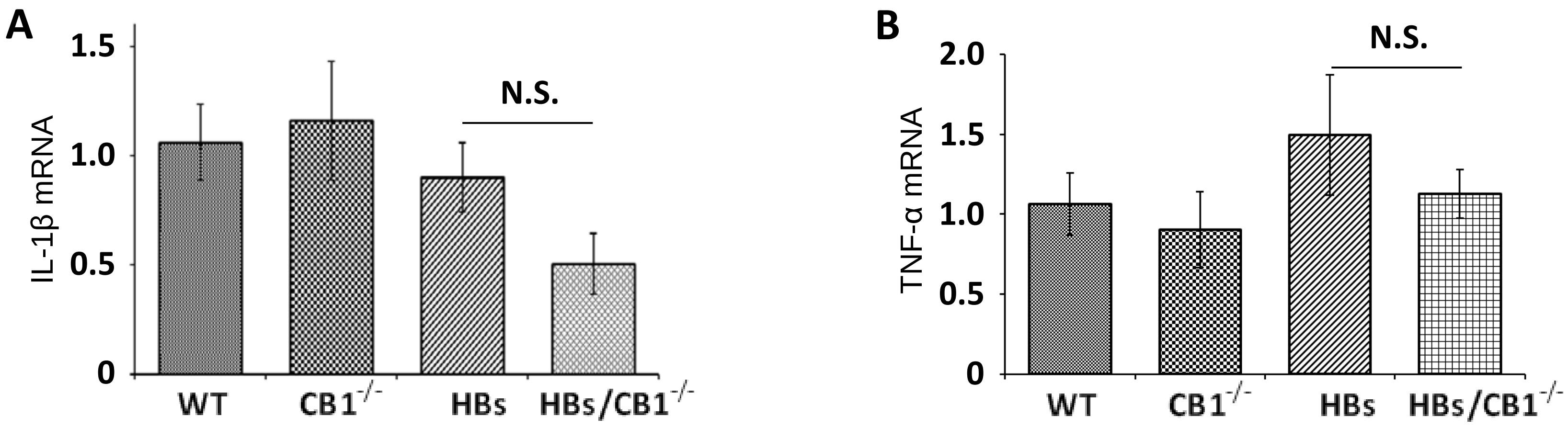

Suppl. Fig. 1: (A and B) qRT-PCR analysis of IL-1β and TNF-α revealed no significant differences between the groups.

Supplemental figure S2: Fibrosis

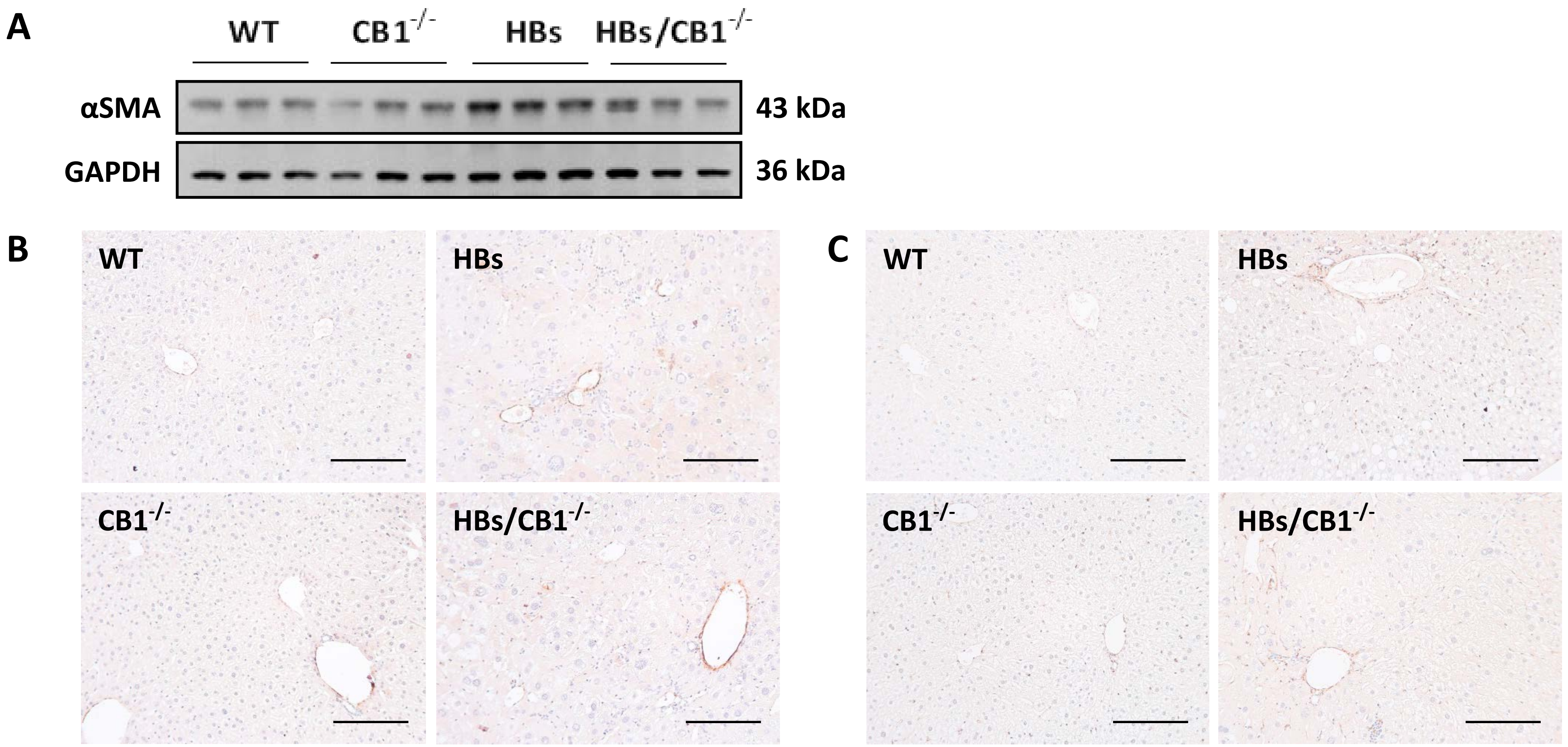

Suppl. Fig. 2: (A) Representative Western blot analysis of lysates from the liver of 52-week-old mice was performed using specific anti-αSMA antibody. αSMA expression is enhanced in HBs transgenic mice in comparison to WT and CB1$^{-/-}$ and at least normalized by trend in HBs/CB1$^{-/-}$ mice. Equal protein loading was confirmed using GAPDH antibody. **(B and C)** Representative immunohistochemical analysis of paraffin-embedded liver sections from 52-weeks old mice were performed using anti-αSMA **(B)** and anti-desmin **(C)** antibodies. Original magnification 200x, bars 200µm.

Supplemental figure S3: Protein expression of PLIN3-5

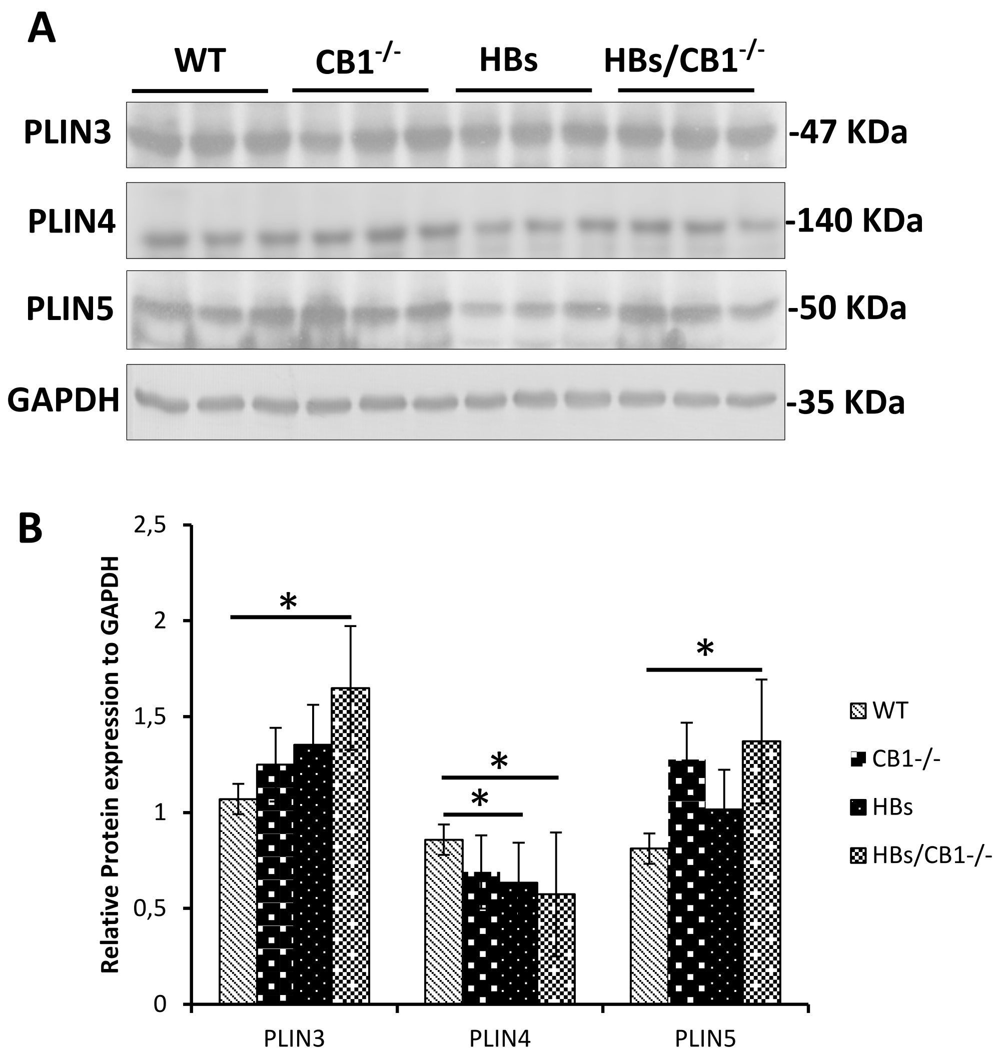

Suppl. Fig. 3: (A) Representative Western blots of hepatic PLIN3, PLIN4, PLIN5, and GAPDH for loading control.

(B) Densitometric analysis of the Western blots given in (A).

Supplemental figure S4: Densitometric analysis of LC3B expression and temporal expression profile

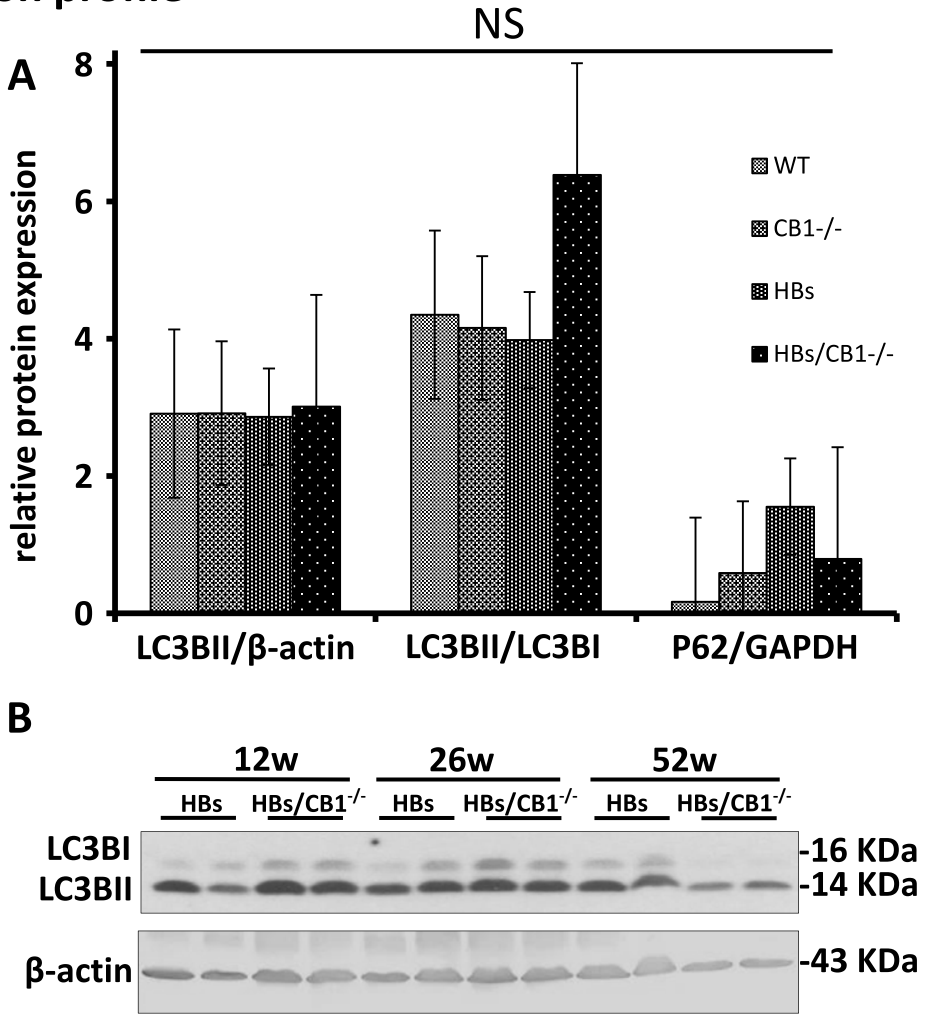

Suppl. Fig. 4: (A) Densitometric analysis of the Western blots given in Fig. 4C. **(B)** Comparative Western blot analysis of LC3B protein expression was performed using two representative liver lysates from 12-, 26- and 52-weeks old HBs and HBs/CB1$^{-/-}$ mice. Equal protein loading was confirmed using anti-β-actin antibody.

Supplemental figure S5: Expression and activation profiles of GLUT1, GLUT2, AMPK, and PPARγ

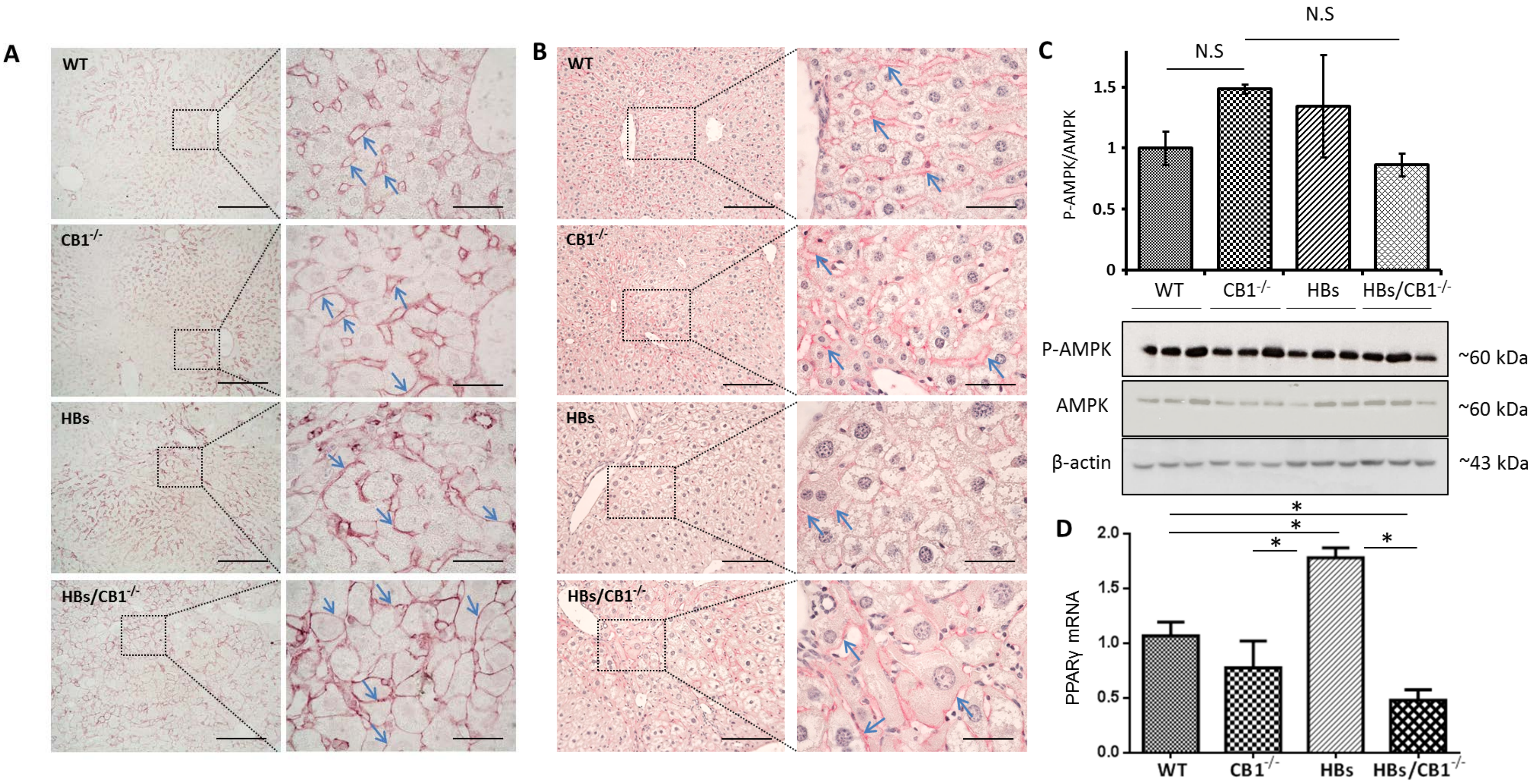

Suppl. Fig. 5: (A&B) Representative immunohistochemical analysis of paraffin-embedded liver sections from 52-weeks old mice were performed using anti-GLUT1 **(A)** and anti-GLUT2 **(B)** antibodies. Images on the right side are the enlarged areas from the boxed areas in the images on the left. Original magnification 200x (left) and 1000x (right), bars 200μm (left), 40μm (right). **(C)** Western blot analysis of lysates from the liver of 52-weeks-old mice was performed using specific anti-phospho-AMPK antibody. Similar level of AMPK expression was confirmed using anti-AMPK antibody. Equal protein loading was confirmed using anti-β-actin antibody. **(D)** qRT-PCR analysis of PPARγ revealed downregulation of PPARγ in $CB1^{-/-}$ mice.

Supplemental figure S6: Schematic summary

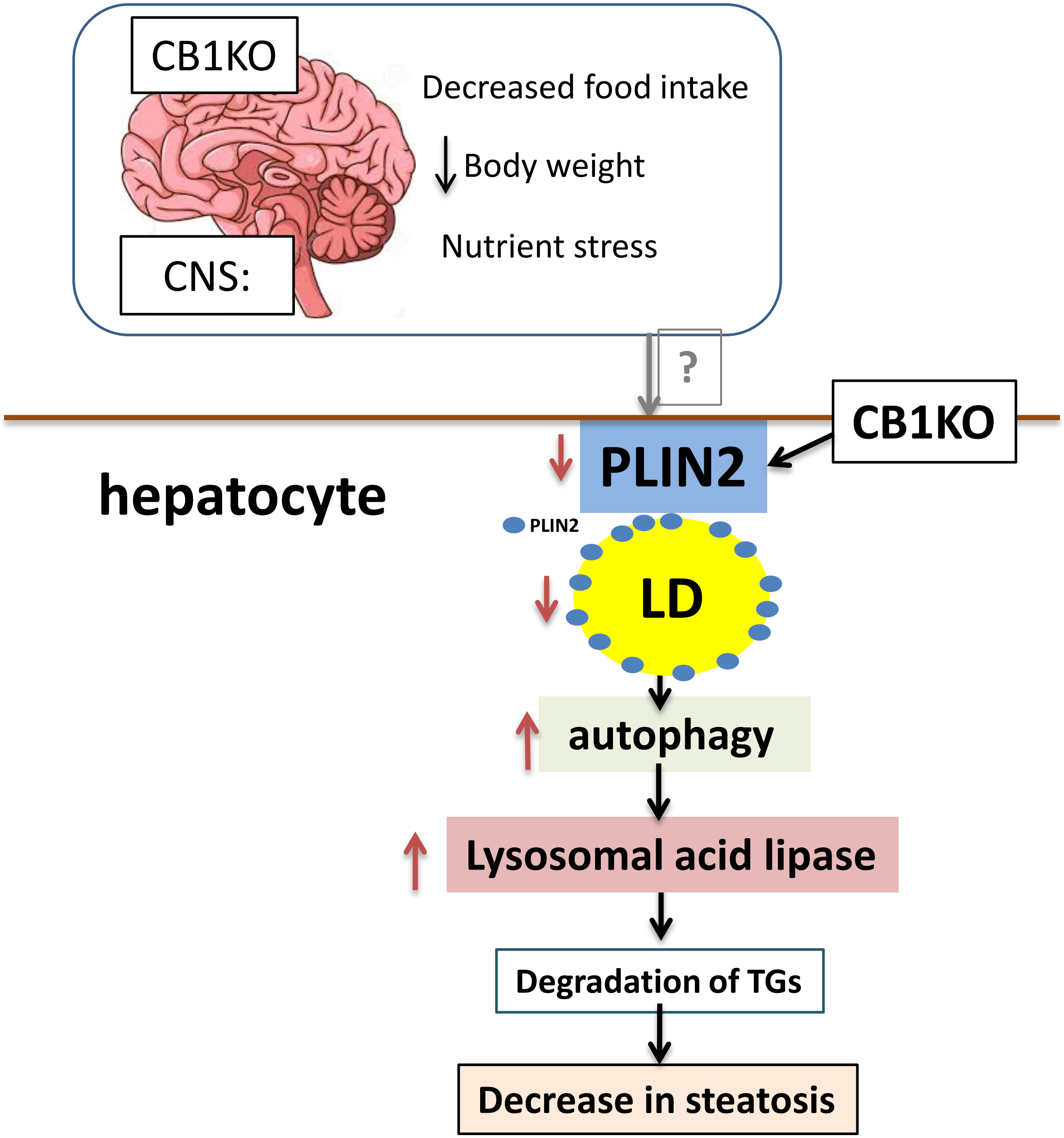

Figure 6: Schematic Summary: Loss of CB1 signaling alleviates hepatic steatosis in HBs transgenic mice via down regulation of PLIN2. $CB1^{-/-}$ causes down regulation of PLIN2 protein expression in in hepatocytes of HBs transgenic mice (model for steatosis). Decreased PLIN2 expression enhances autophagy and autophagic mediated acid lipolysis of lipid droplets i.e. lipophagy. Together, autophagy and autophagic mediated acidic lipolysis cooperate in enhancing the hepatic lipids turnover in HBs transgenic mice. Additionally, the global CB1 receptor knockout mediates decrease energy intake and subsequent reduction in body weight which might contribute to the reduction in hepatic steatosis. In sum, the loss of CB1 signaling alleviates fatty liver via regulation of PLIN2 in the liver of HBs transgenic mice.
